# A comparison of the effect of intranasal desmopressin and intramuscular hyoscine N-butyl bromide combination with intramuscular hyoscine N-butyl bromide alone in acute renal colic

**Published:** 2010

**Authors:** Abdol-Reza Kheirollahi, Mohammad Tehrani, Mohammad Bashashati

**Affiliations:** a Urology Department, Shohadaye-Ashayer Hospital, Lorestan University of Medical Sciences, Khorram Abad, Iran

**Keywords:** Calculus, Renal Colic, Analgesia, Desmopressin

## Abstract

**BACKGROUND:**

Patients with acute renal colic usually require immediate diagnosis and treatment. In this clinical trial analgesic effect of hyoscine N-butyl bromide and desmopressin combination in comparison with hyoscine N-butyl bromide alone in patients with acute renal colic induced by urinary stones was assessed.

**METHODS:**

The study included 114 patients randomly allocated in two groups (A and B). Patients in group A received 20 mg intramuscular hyoscine N-butyl bromide at admission time and patients in group B received 20 μg of intranasal desmopressin in combination with 20 mg intramuscular hyoscine N-butyl bromide. A visual analogue scale (VAS; a 10-cm horizontal scale ranging from “zero or no pain” to “10 or unbearable pain”) was hired to assess the patients’ pain severity at baseline, 30 and 60 minutes after the treatments.

**RESULTS:**

On admission, the pain level was similar in both groups (group A: 8.95 ± 0.11 and group B: 8.95 ± 0.12). In group A, the mean of pain level showed a decrease after 30 minutes (group A: 7.26 ± 0.25 and group B: 5.95 ± 0.28) but further decreasing did not occur; however in group B, the pain consistently decreased and the mean after 60 minutes was significantly decreased (group A: 6.80 ± 0.31 and group B: 3.71 ± 0.31). No side effects were detected in this study.

**CONCLUSIONS:**

The combination of hyoscine N-butyl bromide and desmopressin is more effective than hyoscine N-butyl bromide alone in patients with renal colic. Further studies are recommended to validate these findings and compare the different doses of desmopressin.

Acute renal colic is one of the most agonizingly painful events of a person’s life. Most active emergency wards treat at least a patient with acute renal colic per day. Patients with acute renal colic are often seen and evaluated by emergency physicians at the beginning. Immediate initial treatment besides proper diagnosis and consultations are among the duties of the emergency physicians.^1^,^2^

1-desamino-8-arginine vasopressin (desmopressin) is a vasopressin analogue with a potent antidiuretic activity and less pressor effects in comparison with vasopressin. Hyoscine N-butylbromide is an anticholinergic and antispasmodic agent which is routinely used for patients with acute renal colic.^3^–^5^

According to the previous studies approximately 50 percent of patients who have been treated with intranasal desmopressin had complete relief/reduction of their acute renal colic pain.^1^,^3^ The usefulness of hyoscine N-butylbromide in the treatment of renal colic is not confirmed yet. Studies reported the response rate of 0 to 20 percent for hyosicne N-butylbromide in the treatment of renal colic.^4^–^6^

Intranasal desmopressin may be a reasonable treatment for patients with acute renal colic however further studies are necessary to establish its place.^7^,^8^ To our knowledge, there is no publication on the effect of intranasal desmopressin and intramuscular hyoscine N-butyl bromide combination in renal colic.

Due to low cost, ease of administration, and low adverse effects of desmopressin and hyoscine in comparison with morphine extracts and nonsteroid anti-inflammatory drugs,^1^ this study was conducted to compare the analgesic effect of hyoscine N-butylbromide and desmopressin combination with hyoscine N-butylbromide alone in patients with acute renal colic.

## Methods

This open-labeled clinical trial had been approved by Ethics Committee of Lorestan University of Medical Sciences. Written informed consent was obtained from the patients or their relatives while they were being admitted. The sample size was calculated by considering the effect of desmopressin (instead of desmopressin and hyoscine N-butylbromide combination) and hyoscine N-butylbromide equal to 50 and 20 percent, respectively (□ = 0.05, β = 0.1).^1^–^3^,^7^

Patients who were 18 to 55 years old with clinically diagnosed acute renal colic and without pregnancy, addiction, and any history of hypertension, cardiac insufficiency, surgery on kidneys or ureters, receiving any analgesics/intravenous fluid therapy just before admission, and history of any drug reaction to hyosicne N-butylbromide at emergency ward of Shohada-e-Ashayer hospital (in Khorramabad city, the west of Iran) were randomly divided into two different treatment groups, using a simple randomization method. A shuffle deck of cards (n = 116) provided before the initiation of the study. Half of the cards labeled A (group A) and the other half labeled B (group B). For including a patient, a card was randomly taken from 116 shuffled cards. The taken cards were not returned back to the deck. As a result after inclusion of 116 patients in the study, 58 patients were randomly allocated to each treatment group.

Group A received 20 mg intramuscular hyoscine N-butylbromide (Osveh Pharma. Co., Tehran, Iran) at admission time. Group B received 20 μg of intranasal desmopressin (Minirin, Ferring, Kiel, Germany) combined with 20 mg intramuscular hyoscine N-butylbromide.

A visual analog scale (VAS) score (scored from 0-10) at 0, 30, and 60 minutes of drug administration was utilized to assess the severity of patients’ pain. In each time point, the emergency physician showed the printed VAS line to the patients and asked them to show the number represents their perception of their current pain.

During the study, if a patient could not bear the pain and did not want to continue as a study sample he/she was excluded and administered morphine.

Quantitative data was presented as mean ± standard error of mean (SEM). Repeated measurements ANOVA followed by Bonferroni’s Post-hoc test, independent sample t test and chi-square tests were used in order to analyze the findings.

## Results

The study included 116 patients randomly divided into two different groups (Table 1). Two patients did not continue the study, due to non-tolerable pain.

**Table 1:** Features of patients with renal colic in study groups

	Group A (hyoscine N-butyl bromide) (n = 58)	Group B (hyoscine N-butyl bromide plus desmopressin) (n = 58)	P value
Excluded cases	1	1	-
Age (year)*	31.1 ± 1.1	30.3 ± 0.53	0.4
Male (%)	38 (67)	45 (79)	0.1
Hx of previous passage of urinary stone (%)	11 (19)	7 (12)	0.3
Duration of pain (hour)*	2.5 ± 0.1	2.8 ± 0.17	0.9

* Mean ± SEM

On admission, the mean pain level of patients in group A and B was 8.95 ± 0.11 and 8.95 ± 0.12, respectively (independent t test: p = 0.4).

After 30 minutes the pain decreased in 34 patients (60%) of group A and 49 patients (86%) of group B (chi square: p = 0.001). At this time the mean of pain level of patients in group A and B was 7.26 ± 0.25 and 5.95 ± 0.28, respectively (independent t test: p = 0.001).

In comparison with the time of admission and 30 minutes after it, at 60 minutes after drug administration all patients (100%) in group B revealed pain decreasing, however 11 patients (19%) in group A never showed any pain decreasing (chi square: p = 0.0004). At this time mean of pain levels in group A and B were equal to 6.80 ± 0.31 and 3.71 ± 0.31, respectively (independent t test: p = 0.001).

**Figure 1:**
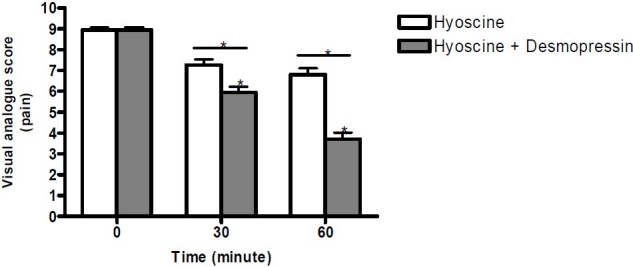
Pain visual analogue score (VAS) in patients with acute renal colic * p ≤ 0.005

In both groups during one hour follow-up, there was a decrease in pain levels but in desmopressin plus hyoscine N-butylbromide group (group B) the trend of pain score decreasing kept its consistency, and after 60 minutes the pain score significantly was less than 30 minutes however in the hyoscine N-butylbromide group (group A) the score at 60 minutes was not less than 30 minutes (repeated measurements ANOVA: group A: p = 0.07, group B: p = 0.005) (Figure 1). No patient showed any side effects.

## Discussion

Patients with renal colic usually require immediate diagnosis and treatment.^2^,^4^ Both non-steroidal anti-inflammatory drugs and morphine have routinely been used for pain control in patients with acute renal colic.^1^,^3^,^8^ Many side effects may arise from these drugs. Narcotic analgesics can induce adverse effects such as sedation, respiratory depression, constipation, addiction, nausea, and vomiting. Moreover non-steroidal anti-inflammatory drugs (NSAIDs) are not harmless and safe in peptic ulcer disease, renal failure, or recent GI bleeding.^9^ In addition, NSAIDs can induce renal failure due to interstitial nephritis.^10^ Therefore, new agents with fewer side effects are of research interest.

Along with pain relieving agents, some physicians use diuretics besides hydration for patients with renal colic. This idea may traditionally arise from using hydration and diuretics to assist stone passage. But some experts concern about the increased hydrostatic fluid pressure in the obstructed urinary tract which can potentially exacerbate patients’ pain.^1^,^9^ Increased hydrostatic pressure proximal to the stone and possible stone migration, increased ureteral peristalsis, tilting of the stone, and intermittent obstructions are the main reasons of pain exacerbation in over-hydrated patients.^1^,^3^ Therefore, it seems that using antispasmodic agents in combination with drugs which minimize hydrostatic pressure of urinary system may be useful in patients with renal colic.

Antimuscarinic drugs including hyoscine N-butylbromide are used for the treatment of smooth muscle spasm. In the genitourinary system the autonomic nervous system is involved in the regulation of ureteric activity by controlling smooth muscle contractility and peristalsis.^9^,^11^ A recent study showed that hyoscine N-butylbromide decreases human ureteric activity to some extent.^9^ Although antimuscarinic drugs are associated with several adverse effects such as photophobia, facial flushing, dry mouth and skin, loss of accommodation, urinary retention and urgency, and constipation^9^; they seem to be safer than opium extracts or nonsteroidal antiinflammatory drugs.

In a study which compared effect of hyoscine N-butylbromide with placebo for patients with stone related renal colic, Holdgate et al demonstrated that hyoscine N-butylbromide does not reduce opioid requirements or the need for ongoing opioid analgesia.^5^ On the other hand some studies revealed that using antimuscarinics decreases pain compared to placebo.^6^,^12^,^13^

Comparing mean intraureteral pressure in two groups of animals showed a significant reduction in pressure following an acute obstruction in subjects treated with desmopressin.^1^,^14^ Desmopressin possibly works by reducing the intraureteral pressure, but it may also directly relax the renal pelvic and ureteral musculature.^1^,^3^ A central analgesic effect through the release of hypothalamic beta-endorphins has been proposed but remains unproved. But up to now the most proper mechanism has not recovered yet. It is not clear whether desmopressin affects renal function or it facilitates stone passage.^3^

Several studies have showed that desmopressin can reduce pain in patients with acute renal colic and the response to the desmopressin is not a placebo effect.^3^,^15^–^17^ Moreover, desmopressin relieves pain quickly with no apparent side effect and reduces the need for other analgesic medications, and can be the only therapy necessary for some patients.^1^ Interestingly, in the present study 20 μg of intranasal desmopressin significantly decreased patients’ pain. Therefore, compared to the previous studies which used 40 μg desmopressin, a good effect was observed. However, parallel comparison of the two recommended doses are recommended for future research.^3^,^15^–^17^

The present study showed that hyosine N-butylbromide either alone or in combination with desmopressin was effective in patients with renal colic. The analgesic effect of the combination was more significant.

## Conclusions

Desmopressin in combination with hyoscine N-butylbromide appears to be a promising alternative or adjunct to analgesic medications in patients with acute renal colic, especially in patients in whom narcotics cannot be used. Further studies on different doses of desmopressin and the parameters which can possibly identify the subgroup of patients who respond better to this medication are needed.
